# Supplementary material for: Smart testing and critical care bed sharing for COVID-19 control
Source: PLoS One. 2021 Oct 6;16(10):e0257235. doi: 10.1371/journal.pone.0257235 (PMC8494319; doi:10.1371/journal.pone.0257235)
Supplement: S1 File — (PDF) [file pone.0257235.s001.pdf]

Supplementary Note 1: Flowchart

The computational platform relies on data given by the user, organized in several input files. In general lines, the required information refers to SEIR parameters (incubation and infected times, initial reproduction rate); basic city data (initial SEIR state of the population, population of the city, hospital capacity); a mobility matrix (having as entries the percentage of population of city i commuting daily to city j); and daily historical records on confirmed infected cases and ICU usage. With the given data, the platform calibrates a time series to forecast the fraction of infected individuals that will need ICU beds every day. Regarding tests, the user must provide the efficiency and the number of days it takes for an infectious to seek assistance (τ). Caps on the testing protocol are also informed by the user (for the number of tests per confirmed cases that are to be performed daily, for logistic limits in each city or in the region). Finally, several targets can be specified in the objective function. For instance for the desired recovered compartment at the end of the horizon, or to promote and/or to discourage restrictions on circulation in a given region.

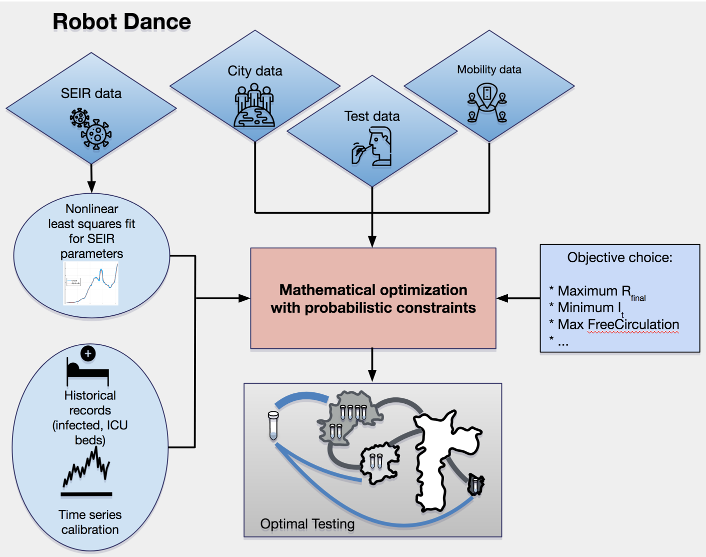

Fig S1.1. Computational framework of the proposed optimal testing and critical care bed sharing for Covid-19 control.

All relevant data used to design a smart testing strategy for Sao Paulo and New York can be found at `https://github.com/pjssilva/Robot-dance`
