# Supplementary material for: Smart testing and critical care bed sharing for COVID-19 control
Source: PLoS One. 2021 Oct 6;16(10):e0257235. doi: 10.1371/journal.pone.0257235 (PMC8494319; doi:10.1371/journal.pone.0257235)
Supplement: S2 File — (PDF) [file pone.0257235.s002.pdf]

Supplementary Note 2: Model Calibration

We developed a computer code named Robot Dance to implement and solve the optimization problem described in the main body of the manuscript. It is written in Julia [1] using the JuMP modeling language [2]. The source code, data, and scripts used in our experiments are available in `https://github.com/pjssilva/Robot-dance`.

The code estimates the initial conditions of the model, namely $(\mathcal{S}, \mathcal{E}, \mathcal{I}, \mathcal{Q}, \mathcal{R})(\mathbb{T}_0)$ using the classical approach of fitting observed data in a time window prior to the simulation period. Specifically, starting from the beginning of the pandemic (without infectious, only susceptible), a SEIQR model gives the evolution of the disease until time $\mathbb{T}_0$. A nonlinear regression then minimizes the error between the historical data and the SEIQR model. One important caveat is that the historical records span over a range of times in which different social distancing and other containment measures were put in place. This means that the model must consider time varying reproduction numbers R_t in the SEIQR relations. Since those values are unknown, in addition to the conditions at $\mathbb{T}_0$, the calibration estimates associated values for R_t, for each day of the history of records. The estimation is done by endowing the Robot Dance code with an objective function that combines least-square error measurements with a total variation regularization [3] to avoid overfitting.

The history of observed data was retrieved from official sources from the state of Sao Paulo [4]. In the case of New York city we used records from [5]. As pointed out in [6] and [7], the number of COVID-19 cases is underreported both in Brazil and the US. We corrected the number of cases in the state of Sao Paulo using information from a serological inquiry made in the whole Sao Paulo city [8]. For New York city we performed a similar analysis correcting the number of cases in order to have 80% of susceptibles on April 23, 2020, as suggested in [9].

Suppose the data is available for p days before $\mathbb{T}_0$, and consider the time period $[\mathbb{T}_0 - p, \mathbb{T}_0]$. Then, for each area i individually, given the corrected number of new cases $\bar{\mathcal{I}}_0^i, \bar{\mathcal{I}}_1^i, \ldots, \bar{\mathcal{I}}_p^i$, the calibration consists in solving the optimization problem

$$\left\{ \begin{array}{lll} \displaystyle\min_{R_t^i \in [0, R_0]} & \displaystyle\sum_{t \in [\mathbb{T}_0 - p, \mathbb{T}_0]} (\mathcal{I}_t^i - \bar{\mathcal{I}}_t^i)^2 + & \displaystyle\nu \sum_{t \in [\mathbb{T}_0 - p + 1, \mathbb{T}_0]} |R_t^i - R_{t-1}^i| \\ \text{s.t.} & (\mathcal{S}_t^i, \mathcal{E}_t^i, \mathcal{I}_t^i, \mathcal{Q}_t^i, \mathcal{R}_t^i, R^i) & \text{satisfies the discretized SEIQR equations,} \end{array} \right. \tag{1}$$

and where ν is a parameter that controls the effect of the total variation regularization. We used $\nu = 0.25$ that showed to induce a good balance between the curve fit and the stability of the reproduction number R_t. The optimization includes historical data on daily tests, if available (the number of daily tests is set to zero, otherwise). Notice that since calibration is performed separately for each area i, mobility is not taken into account in the estimation phase.

The procedure outputs $(\mathcal{S}_t^i, \mathcal{E}_t^i, \mathcal{I}_t^i, \mathcal{Q}_t^i, \mathcal{R}_t^i)$ and R_t^i for $t \in [\mathbb{T}_0 - p, \mathbb{T}_0]$. The final values $\mathcal{S}_{\mathbb{T}_0}^i, \mathcal{E}_{\mathbb{T}_0}^i, \mathcal{I}_{\mathbb{T}_0}^i, \mathcal{Q}_{\mathbb{T}_0}^i, \mathcal{R}_{\mathbb{T}_0}^i$ yield the desired initial conditions for the simulation in the interval $[\mathbb{T}_0, \mathbb{T}_1]$.

References

1. Bezanson J, Edelman A, Karpinski S, Shah VB. Julia: A fresh approach to numerical computing. SIAM review. 2017;59(1):65–98.

2. Dunning I, Huchette J, Lubin M. JuMP: A Modeling Language for Mathematical Optimization;59(2):295–320. doi:10.1137/15M1020575.

3. Chambolle A. An Algorithm for Total Variation Minimization and Applications. Journal of Mathematical Imaging and Vision. 2004;20(1):89–97. doi:10.1023/B:JMIV.0000011325.36760.1e.

4. URLdata-online;. Available from: https://www.seade.gov.br/coronavirus/.

5. URLdata-online;. Available from: https://github.com/nychealth/coronavirus-data.

6. Bastos SB, Morato MM, Cajueiro DO, Normey-Rico JE. The COVID-19 (SARS-CoV-2) Uncertainty Tripod in Brazil: Assessments on Model-Based Predictions with Large under-Reporting. Alexandria Engineering Journal. 2021;60(5):4363–4380. doi:10.1016/j.aej.2021.03.004.

7. Noh J, Danuser G. Estimation of the Fraction of COVID-19 Infected People in U.S. States and Countries Worldwide. PLOS ONE. 2021;16(2):e0246772. doi:10.1371/journal.pone.0246772.

8. URLdata-online;. Available from: https://www.prefeitura.sp.gov.br/cidade/secretarias/upload/saude/17_9_2020_PPT_COLETIVAADULTO_FASE%205.pdf.

9. URLdata-online;. Available from: https://www.nytimes.com/2020/04/23/nyregion/coronavirus-antibodies-test-ny.html.
