# Supplementary material for: Smart testing and critical care bed sharing for COVID-19 control
Source: PLoS One. 2021 Oct 6;16(10):e0257235. doi: 10.1371/journal.pone.0257235 (PMC8494319; doi:10.1371/journal.pone.0257235)
Supplement: S3 File — (PDF) [file pone.0257235.s003.pdf]

Supplementary Note 3: New York City

For NY city, taking data from [1] we analyzed the pandemic evolution for the period between April 14, 2020 and May 20, 2020. During this period tests were done but no contact tracing, the setting modeled by Robot Dance, the computational platform described in the Supplementary Note 1. (according to `https://www.nytimes.com/2020/06/21/nyregion/nyc-contact-tracing.html` the contact tracing protocol was put in place in June 1, 2020). Since for this study the focus is on the number of lives that are protected by a specific testing protocol. the platform maximizes the final number of susceptible individuals (preserved from the infection, partly thanks to testing).

The benchmark was done under the following premises: the initial conditions and the R_t profile for the whole period was obtained as described in the Supplementary Note 2. Our estimation incorporated available data of historical records of daily tests. As a result, the calibrated value R_t accounts for a situation that excludes from the model the effect of testing. We also used testing efficiency of 80% and $\tau = 2$. We considered the following instances.

1. Actual: the test pattern that was actually applied in the city for the considered period.

2. Smart: the optimal test distribution computed by Robot Dance, using the same budget of tests that were available for the period in case 1.

3. Extra-budget: Robot Dance optimal distribution, should the testing capability be increased ten times. This instance represents the ideal scenario.

Our analysis sheds a light on some important issues. Testing helps in controlling the spread of the disease, and does save lives. According to our calculations, and rounding up, the figures are the following. The testing actually applied in NY city for the considered period (option Actual) spared from the infection 15767 individuals. Applying Robot Dance protocol (option Smart) would have spared 10315 more individuals, that amounts to 10315+15767=26082 saved lives. This represents an increase of 65% more protected individuals, that is achieved using the same amount of tests than in 1, but distributing them in a more efficient manner along time. A tenfold increase in the number of tests (option Extra-budget) results in additional 21670 preserved lives. With respect to option Actual, this means that 26082+21670=47752 more individuals would have been kept infection free.

The efficiency of testing depends not only on the number of tests performed daily, but also on when the tests are done. This phenomenon is clear when comparing Actual and Smart, as both options employ the same number of tests, but conduct them at different times. Figure S3.1 shows on the left the percentage of ICU capacity that is occupied and on the right, the time at which tests were conducted for each variant.

References

1. URLdata-online;. Available from: `https://github.com/nychealth/coronavirus-data`.

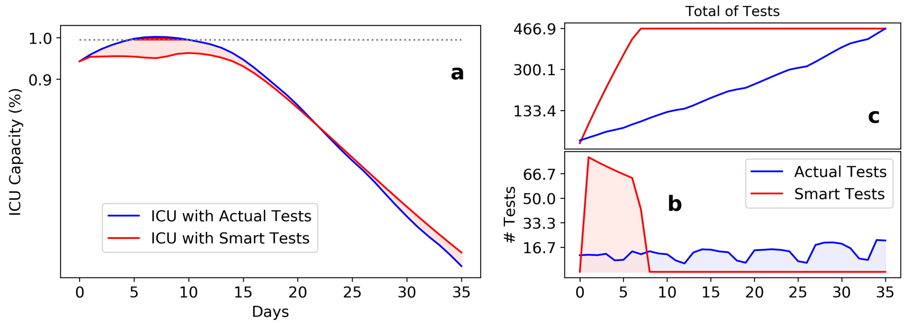

Fig S3.1. ICU occupancy and testing in NY city. On the left, (a) compares the ICU use with Actual and Smart testing protocols. Notice that the actual testing resulted in a saturation of the hospital capacity that lasted some days (the thin red area over the dotted horizontal line). By contrast, smart testing succeeds in keeping the use of critical beds below the maximum available. This is explained by the right plots, the daily distribution of tests in (b) shows that the smart option performs all the tests at the beginning of the period whereas the actual testing was deployed uniformly along time. As reported in (c), both options conducted the same number of tests, but the timing of smart testing was more successful.
