# Supplementary material for: Smart testing and critical care bed sharing for COVID-19 control
Source: PLoS One. 2021 Oct 6;16(10):e0257235. doi: 10.1371/journal.pone.0257235 (PMC8494319; doi:10.1371/journal.pone.0257235)
Supplement: S4 File — (PDF) [file pone.0257235.s004.pdf]

Supplementary Note 4: Additional scenarios in Sao Paulo state

We discuss here some additional figures and results of interest, exploring to which extent testing helps avoiding lockdowns. The same premises are the same than in Methods of the main manuscript, that is $R_0 = 1.8$, $\tau = 3$, and circulation is encouraged while respecting the hospital capacity. The aim is to maximize the number of recovered individuals at the end of a 390 day period starting on July 1, 2020. We compare the pandemic evolution for the three macro-regions that compose Sao Paulo city, metropolitan area, and interior/rural area, under different testing protocols specified below.

1. Test-XXK: Testing starts on day 140. From this day on, there are no restrictions on the circulation. The budget of tests is XXK$\in \{30, 100\}$ thousand per day. For Sao Paulo state, these ranges amount to conducting $\{750, 2500\}$ daily tests per million of inhabitants.

2. Smart-XXK: Testing XXK starts on day 1, circulation is restricted only if hospital capacity is close to saturation.

In order to assess if none or one single measure of containement would suffice to contain the spreading, we also analyzed the following two extreme scenarios.

3. Smart-00K: There is no testing, but some level of confinement is imposed when needed.

4. DoNothing: No test and no confinement imposed. This scenario represents an absence of any public measure.

The comparison of the extreme options with Test-XXK and Smart-XXK is shown in Figure S4.2 for the hospitals of the three macro-regions. In general, testing alone fails in keeping the epidemiological situation under control outside of Sao Paulo city.

Test distribution

The distribution of tests in the three macroregions is shown in Figure S4.1, where insets (a,b) correspond to Test-XXK while insets (c,d) correspond to Smart-XXK.

ICU occupancy

Recall the difference pointed out near day 200 between Figure S4.1 (a) and (c). The explanation for Test-30K (a) to perform tests in the metropolitan area instead of Sao Paulo city, as Smart-30K (c) does, lies in the ordinate values in Figure S4.2. Namely, in the metropolitan area (Figure S4.2 (b), the hospital occupancy is close to 80% of the capacity, while in Sao Paulo city only 60% of the beds are used (a). In order to prevent a saturation of the hospital capacity, more testing is conducted in the metropolitan area and, since the daily limit is low (30K), this leaves Sao Paulo city without any tests. The opposite action taken by Smart-30K, which tests more in Sao Paulo city (Figure S4.1 (c)), is possible because during that period the circulation in the metropolitan area is restricted, due to confinement. Since Test-30K does not consider restricting circulation, it enters the red area in (b) of Figure S4.2. We conclude that a strategy of confinement

with testing (Smart-30K) or without testing (Smart-00K) is more effective than only testing, at least in a configuration close to hospital saturation.

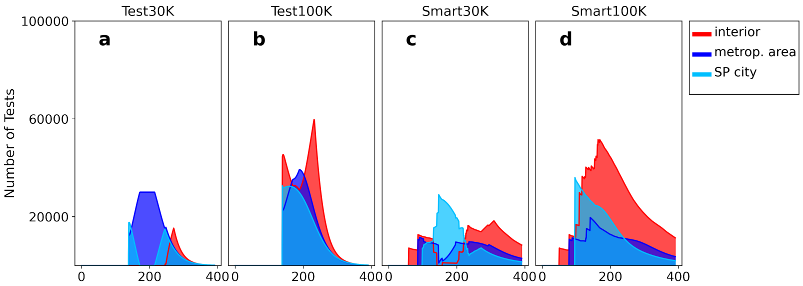

Fig S4.1. Option Test-XXK, testing from day 140 on without limiting the circulation, insets (a,b): if the daily capacity is low (a), testing is mostly done in the metropolitan area and to some extent in Sao Paulo city, but not in the interior. When comparing the red areas in (a) and (b) notice that early testing the interior is only done if the capacity climbs up to 100K daily tests; otherwise, testing starts in this subregion much later. This shift is explained by the different epidemiological time of the interior, where the virus started circulating later. Sao Paulo city, with its international airports, was the initial epicenter from which the infection progressively spread into the full state. Option Smart-XXK, insets (c,d): tests less in the metropolitan region (dark blue areas in c,d are smaller than in a,b) and more in the interior (red areas in c,d are larger than in a,b). When the testing capacity is low, around day 200, the smart option (c) assigns all the tests to Sao Paulo city, while the alike instance (a) performs the tests in Sao Paulo metropolitan area instead.

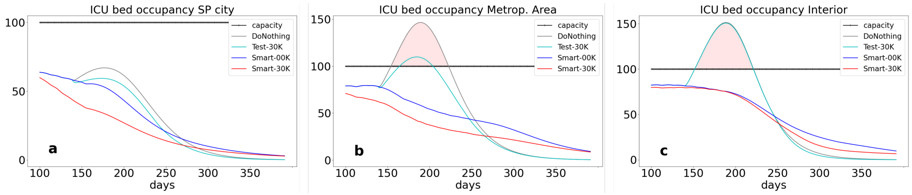

Fig S4.2. Percentage of ICU bed occupancy for (a) Sao Paulo city, (b) metropolitan area, and (c) interior. The downwards tendency in SP city (a) indicates that an epidemiological peak was left behind in the region. The idle hospital capacity in SP city is about 40%, so doing nothing or performing only tests seem acceptable strategies. For the other regions the situation is substantially different. The metropolitan area population is as large as Sao Paulo city's, but has much less health infrastructure (472 ICU beds instead of 3434 in Sao Paulo city). Doing nothing or only performing tests plunges the region into a catastrophic epidemiological situation (b), the surge of hospitalizations would need an increase in the total capacity of about 40%, for over two months (approximately days 170-230). Testing makes the situation less bad, but the improvement is not sufficient to address the critical situation, the health system still cannot bear the surge in hospitalizations. By contrast, only with confinement (Smart-00K) there is no collapse, and adding testing campaigns (Smart-30K) reduces further the hospitalizations. Similarly for the interior (c), doing nothing or just testing drives the health system to almost two months with a deficit of up to 50% of ICU beds. The region population doubles Sao Paulo city's, but has 403 ICU beds instead of 3434 in Sao Paulo city.

Recovered compartment

The time lag of almost two months between the pandemic situation in Sao Paulo city and the rest of the state can be measured by comparing the ordinates of the graphs in Figure S4.3, with the percentage of individuals that are in the recovered compartment at the end of the horizon. At day 100 Sao Paulo city has more than 30% of its population in the recovered compartment. A similar level is reached in the interior only about 2 months later (day 150).

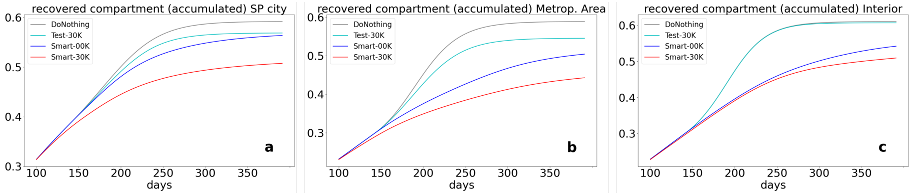

Fig S4.3. Percentage of individuals in the R compartment for (a) Sao Paulo city, (b) metropolitan area, and (c) interior.

Impact of shorter delays in testing

When hospitals are put under stress, the results in Figure S4.2 suggest restricting the circulation as the only solution to control the pandemic. Notwithstanding, after more than half a year of social-distancing, imposing that type of measures becomes less and less possible, simply because a vast portion of the society stopped adhering to them. For this reason, in an effort to determine when testing can effectively replace confinement measures, we repeated our study, this time assuming the testing delay can be shortened (in the technological procedure itself and/or in the administrative handling of the samples). For example, this could be a new fast technology that is reliable to conduct tests.

For our model, this amounts to setting the delay between symptoms and test results (and possible quarantine) to $\tau = 1$ day. The situation changes significantly, even though peaks beyond the local hospital capacity remain outside of Sao Paulo city. With the realistic cap of 30K tests per day, in the best option of smart testing, both the metropolitan area and the interior have a deficit of beds, illustrated in Figure S4.4.

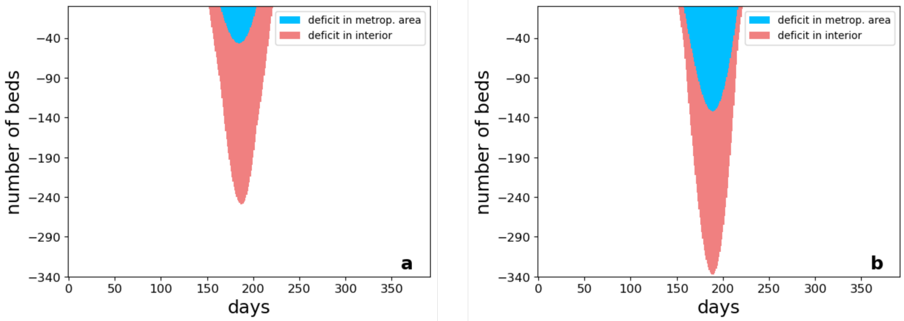

Fig S4.4. Missing beds to attend the surge of hospitalizations in the metropolitan area and interior when applying smart testing with a cap of 30K tests per day. The delay in days between seeking for attention in the hospital and getting the test results is $\tau = 1$ (a) and $\tau = 3$ (b). The critical period with the latter extends for almost two months (approximately days 152 to 211).

Since the interior is behind Sao Paulo city in terms of epidemiological spread, giving

to this region access to part of the idle hospital capacity of Sao Paulo city can be a solution. The positive impact of this measure is illustrated by Figure 3 of the main manuscript. Therein, 500 beds are reserved in Sao Paulo city to create a pool of 134, 106 and 260 beds for Sao Paulo city, the metropolitan area and the interior. The respective changes in the local hospital capacity are -4%, +22%, and +65%. When $\tau = 1$, the pool is sufficient to compensate for the deficit of beds in the interior. In the metropolitan area, the 30K-Test strategy now suffices while formerly it was not enough for the local hospitals to attend the surge by their own (see Figure S4.2(b)). Finally, notice that if the delay is $\tau = 3$ days, Figure S4.4(b) shows that the same pool of 500 beds fails in solving the situation in the state, even with smart testing. When $\tau = 3$, the metropolitan and interior areas would need 140 and 340 additional beds, but they only have access to 106 and 260, respectively. This confirms the interest of developing new technologies of testing, that provide reliable results faster.
