# Supplementary material for: Smart testing and critical care bed sharing for COVID-19 control
Source: PLoS One. 2021 Oct 6;16(10):e0257235. doi: 10.1371/journal.pone.0257235 (PMC8494319; doi:10.1371/journal.pone.0257235)
Supplement: S5 File — (PDF) [file pone.0257235.s005.pdf]

Supplementary Note 5: Effective Models

We also consider a distinct model to capture the effects of testing. Here, we follow Refs. [1, 2]. In this setting, the quarantine is modelled by a delay differential equation and a mathematical analysis provides an effective reproduction number in terms of the time spent to identify the patients $\bar{\tau}$. If infected individuals are isolated after $\bar{\tau}$ days after being infectious, this leads to an effective reproduction R_{eff}. Here we use $\bar{\tau} = 5$ to capture the fact that for two days individuals can infect others but do not exhibit symptoms and then after $\tau = 3$ days with symptoms they are isolated.

$$R^i_{\text{eff}}(t) = R^i(t)(1 - p^i e^{-\bar{\tau}/T_{\text{inf}}}) \tag{1}$$

Here p_i is the probability that a SARS patient have COVID-19 as in the Methods section of the main manuscript. In our optimization, we always consider $R^i_{\text{eff}}(t)\mathcal{I}^i(t)$. The remaining procedures are as before. We will consider a scenario where the state of Sao Paulo has an 1100 daily testing capacity for per million inhabitants.

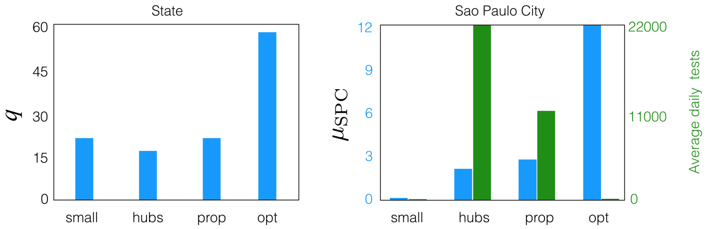

Fig S5.1. Prediction of monthly efficiency of different testing strategies on the control of COVID-19 using effective models. We consider a daily cap in the number of test of 1080 per million inhabits. We compare four scenarios: (a) testing only areas of small population, (b) testing only the hub cities, (c) testing according to demand – the local fraction of infected, and (d) the optimal testing strategy. In the left inset, we show the average monthly efficiency q the testing strategy has with respect to the no test case – using only lockdown as mean of control. All testing strategies lesser the control with respect to social-distancing measures only. However, the smart testing is almost three times more effective. In the right inset, we show the factor q for the main city of Sao Paulo along with the daily average number of tests each strategy sends to Sao Paulo. With respect to the testing on demand, the optimal strategy controls Sao Paulo improves the factor q by threefold while sending 2% of the total amount of tests send there for the on demand strategy. Thus, smart testing leads an effective control allowing for opening of non-essential services and as well as to an sharp increase in the number of inhabitants enjoying the new normal.

Under this daily testing cap we consider different scenarios: testing only in the small local health area, testing only in the hubs, testing according to demand, that is, the number of tests of proportional to the percentage of infected individuals in the local population, and finally, the smart testing. All the first three strategies give roughly the same efficiency $q \approx 20\%$. This highlights the WHO recommendation for a strong testing program as testing does provide major help to mitigation strategies. Strikingly, the optimal strategy is much superior to the others given the efficiency of $q \approx 55\%$.

Smart testing sends on average 270 daily tests per million inhabitants to the city of Sao Paulo as opposed to the testing on demand which sends an average of 11500 daily

tests per million inhabitants. The counter-intuitive effect is that smart testing has near 3 fold improvement in comparison to testing on-demand or testing only the hub local health area, as shown in Figure S5.1.

In smart testing, the relation between the test distribution and improvement in the control also has an intricate spatiotemporal signature. To access this effect, for each day, we compute the Pearson correlation $\rho(t)$ between the efficiency $\mu_i(t)$ in the control and the number of tests $\#\mathrm{T}^i(t)$ per million in the ith local health area. In Figure S5.2 we compare the testing on demand and smart testing. Interestingly there is no significant correlation between local health area's efficiency μ_i and number tests and tests are distributed according to demand as observed in the right inset. In smart testing in the first six months of control, there is a significant correlation between efficiency and tests. This changes after roughly six months when the correlation becomes high. Thus the smart testing explores and strong patio-temporal behavior of the spreading throughout the state.

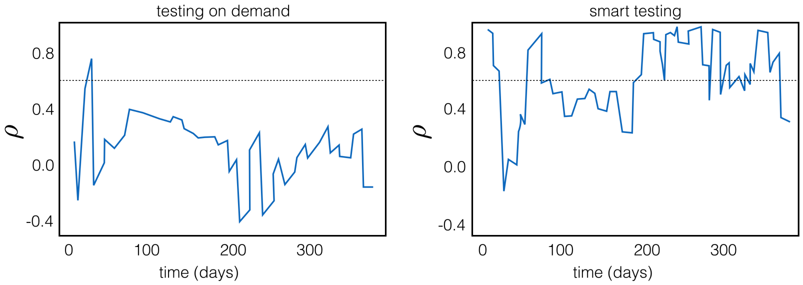

Fig S5.2. Correlation ρ between the number of cases and tests made and efficiency in control for different scenarios. For each day, we compute the correlation between the efficiency $\mu_i(t)$ in the control and the number of tests $\#T$ per million across the local health area's. Surprisingly, when tests according to the demand there is no significant correlation. In smart testing, in the first six months of control, there is no relevant correlation between efficiency and tests. This changes after roughly six months when the correlation becomes significantly high. The dashed line represents the correlation significance level of 5% ($\alpha = 0.05$). Thus the optimal protocol deeply explores and strong patio-temporal behavior of the spreading throughout the state.

References

1. Ruschel S, Pereira T, Yanchuk S, Young LS. An SIQ delay differential equations model for disease control via isolation. Journal of mathematical biology. 2019;79(1):249–279.

2. Young LS, Ruschel S, Yanchuk S, Pereira T. Consequences of delays and imperfect implementation of isolation in epidemic control. Scientific reports. 2019;9(1):1–9.
