# Supplementary material for: Smart testing and critical care bed sharing for COVID-19 control
Source: PLoS One. 2021 Oct 6;16(10):e0257235. doi: 10.1371/journal.pone.0257235 (PMC8494319; doi:10.1371/journal.pone.0257235)
Supplement: Attachment — Submitted filename: Response to Reviewers.pdf [file pone.0257235.s008.pdf]

Imperial College London

Department of Mathematics
Imperial College London
Room 638, Huxley Building
180 Queens Gate
London SW7 2AZ
Tel: +44 (0)207 5948502
Fax: +44 (0)207 5948517
tiago.pereira@imperial.ac.uk
Tiago Pereira, PhD
Professor of Applied Mathematics

16 April 2021

Dear Editor,

Thank you for your letter and the opportunity to revise our paper on ‘Smart testing and critical care bed sharing for COVID-19 control’. We are delighted that two referees recommended the paper for publication and offered suggestions to improve its presentation. The first referee had concerns about the role of asymptomatic as you also pointed out. We have addressed this issue in the new version. We have included the reviewer’s comments immediately after this letter and responded to them individually, indicating exactly how we have addressed all points raised by the referee and Editors.

The major changes are:

i) New references are included
ii) Discussion about the role of asymptomatic.
iii) Further explanations for the model.

Sincerely yours

Tiago Pereira, PhD

Additional Editor Comments:

The main issues to be addressed are the model formulation and providing supporting references to some of the statements and value referred to as being informed by data. The model explicitly account for asymptomatic infection. One of the key parameter to the analysis is the rate at which people are seeking for care, but it is based on data from people with covid-19 like symptoms therefore it does not account for asymptomatic who are less likely to seek care and be tested and isolated. Those asymptomatic individuals will spread the disease for a longer time period that tested symptomatic individuals. By not modeling asymptomatic explicitly, the model is likely to overestimate the effectiveness of the intervention. A Us-based study has shown that symptomatic individuals are 2 to 4 times more likely to be tested for covid-19 than asymptomatic https://www.nature.com/articles/s41562-020-00944-2#Sec4

We thank the referees and the editor for the careful evaluation of our manuscript and for their thoughtful comments. We believe that the input has improved our paper. The manuscript was not sufficiently clear in respect to asymptomatic. Asymptomatic individuals were taken into consideration in the model and in the code. The simulations reported account for this distinction, but the text did not. Thanks for highlighting this important point. We added a discussion in lines 300-310 of the revised version of the manuscript.
As for the rate at which individuals seek care, our implicit modelling approach of asymptomatic individuals does not incur overestimation. The estimate of ICU bed usage is based on the size of the I compartment as a function of time, which accounts for both symptomatic and asymptomatic individuals. Additionally, we estimate the under-reporting of infected new cases based on serological surveys conducted by health services. This factor considers the asymptomatic individuals.

In addition these comments, please thoroughly address the issues raised by the three reviewers.

We address the issues raised by the referees below.

Review Comments to the Author
Reviewer #1:

The article "Smart testing and critical care bed sharing for COVID-19 control" by Pereira et al. investigates the optimal use of PCR test allocation and ICU bed sharing to mitigate COVID-19 transmission in the São Paulo region of Brazil using a compartmental model of COVID-19 transmission embedded within an optimal control framework. The general methodology and derived results are interesting, however I have serious concerns about the structure of the underlying transmission model. In particular, the model does not seem to capture either asymptomatic or presymptomatic transmission of infected individuals - both of which are understood to contribute substantially to overall transmission. (Whilst in the Methods section a claim is made that the latter is accounted for through the parameter \zeta, I found this claim unconvincing and believe that more natural ways of including this effect exist). Therefore, I believe the base model is unsuitable for the desired application and, as such, I must recommend that this article not be accepted for publication.

> The manuscript was not sufficiently clear in this respect. Asymptomatic individuals were taken into consideration in the model and in the code. The simulations reported account for this distinction, but the text did not. We added a discussion in lines 300-310 of the revised version of the manuscript and rephrased Eq. (14) to highlight this point. The estimate of ICU bed usage is based on the I compartment, which accounts for both symptomatic and asymptomatic individuals. We estimate the under-reporting of infected new cases based on serological surveys conducted by health services. This factor considers the asymptomatic individuals.

In addition to the above, please find further comments below:
- Throughout the text several claims are made without reference

> In the new version of the manuscript we provided the new references [5-20].

- Why did the authors choose to use the R0 value for the second wave in Germany? Surely there is sufficient data from Brazilian cases to estimate the local reproduction number?

> Indeed, we also have this number for Brazil by solving an optimization problem with total variation and it is very close to 1.8. Further details can be found in
> https://www.ime.unicamp.br/~pjssilva/vidas_salvas.html
> We provide this discussion in Pg. 6 of the manuscript.

- Lines 121 - 129: This paragraph is repeated from above

> The paragraph was removed.

- The discussion contains no reflection on the limitations of the modelling framework and analysis

> We added a paragraph in Discussion section lines 245.

- I believe that the interpretation of \lambda as the percentage of infected individuals that are quarantined is incorrect, from equation (10) it appears that the true percentage is \lambda / (1 + \lambda).

> Lambda is a rate. We amended the manuscript.

- In general, I found the methods section difficult to follow with several key parameters left undefined

> We added a paragraph about the protocols (line 315) and details on parameters such as mobility matrices (line 280) and role of asymptomatic (line 310). In the GitHub we also provide a full code and all parameters to perform simulations on smart testing.

Imperial College of Science, Technology and Medicine

Reviewer #2:

The present manuscript analyses the problem of optimising ICU bed occupancy in the current COVID-19 pandemics and the effect of different testing strategies. The paper is nice, relevant and elegant although some details could be the text. I reckon the paper can be useful as case-study even for other stances such as ICU bed sharing in the UE.

We thank the reviewer for the effort spent evaluating the manuscript and for the critical feedback. Below we address the points raised.

Nonetheless, I would like to see some points further discussed.
- The authors mention their study spans the period between 1st July 2020 and 31st July 2021, but today is the 23rd March 2021. is it between 1st July 2019 and 31st July 2020 instead?

The tests were done encompassing the period from July 2020 to July 2021 so we could assess how the different test regimes would affect the future outcome of the pandemic.

- The epidemiological model used in the paper is similar to that used in the following paper for Brazil as well
https://www.worldscientific.com/doi/abs/10.1142/S0129183120501351

The reference was included in the Introduction

- The impact of testing strategies is largely impacted by the so-called asymptomatic cases, who are not prompt to go testing. Models considering such state (eg https://www.nature.com/articles/s41598-020-76257-1 andhttps://arxiv.org/abs/2005.09019)

We explained how the model treats asymptomatic individuals in lines 300-310.

- A mobility model should discuss the economical problem of people who cannot isolate because they need to get income.

We added the discussion (lines 275)

- Further details on the network structure used in the work should be provided. Do authors consider small world, scale-free, multi-layer networks?

The network is obtained from data and its structure is similar to a small-world (line 280).

Reviewer #3:

The authors have presented a very interesting study, combining a rigorous mathematical modelling approach with effective use of data and a discussion of real-world clinical consequences. A novel optimisation approach is used, in which the authors calculate the maximum amount of epidemic growth allowed given limits on specific clinical outcomes; this allows them to propose testing approaches which are effective in reducing morbidity and mortality but also allow for a maximum of "normal" social behaviour.

My main critique of the manuscript is that the smart testing methodology is not sufficiently explained within the main paper. The application of smart testing is the main focus of the paper, but the description of smart testing is limited to a short outline at the start of the results section on Page 4. In particular, this very general account makes it difficult to know exactly how smart testing differs from the on-demand and hub testing strategies. For publication, the authors need to add a few paragraphs to the methods section of the manuscript explaining the principles behind their smart testing approach, and highlighting the contrasts with the other two strategies.

We added a paragraph comparing protocols (line 315) and details on mobility matrices (line 280) as well as role of asymptomatic (line 310). In the GitHub we also provide a full code and all parameters to perform simulations on smart testing.

My other criticisms are fairly minor. Throughout the manuscript, the authors use a lower-case "r" for reproductive ratios. Typically in infectious disease modelling, lower case "r" is reserved for growth rates, with upper case "R" used for reproductive ratios. To improve the clarity of their paper, the authors should bring their notation into line with this convention. Also throughout the paper, the authors hyphenate the phrase "social distancing", writing it as "social-distancing". There is no need for a hyphen here, and a hyphen is not used in, for instance, UK or USA government guidelines on social distancing, and so the authors should replace these hyphens with spaces.

Now capital R stands for reproduction number and calligraphic R for the recovered compartment.

In the abstract, the authors talk about "The economic infrastructure of the country" when talking in general about developing countries. Because they are talking about a group of countries, this should say "The economic infrastructure of these countries".

We implemented it.

Bottom of page 6, line 113: "The model assumes the basic reproductive number with sanitary measures is $r0 = 1.8$ (the value observed for the second wave in Germany [11])." The basic reproductive ratio is highly dependent on the social setting, and given the socioeconomic contrasts between Germany and Brazil (which the authors do an excellent job of highlighting), the value in Germany may not be a useful indicator of the value in Brazil. It is also worth pointing out that reference 11 points to a website where an R0 estimate is not immediately visible! If possible, the authors should find an estimate specifically from Brazil, or if this is not possible try to find a range of R0 values across different socioeconomic settings, and compare their chosen value with this range.

Indeed, we also have this number for Brazil and it is very close to 1.8. We calculated it by solving an optimization problem with total variation. Further details can be found in
https://www.ime.unicamp.br/~pjssilva/vidas_salvas.html
We provide this discussion in Pg. 6 of the manuscript.

From page 6 line 113 to page 7 line 129 we get two phrasings of the same paragraph. This appears to be a proofreading error, and one of these paragraphs should be deleted.

We removed the paragraph.

Page 7, line 134: “When ri_no test(t) = r0, the basal reproduction number of the new normal, there are no travel restrictions.” It is unclear to me what the phrase “new normal” refers to – here the authors appear to mean the situation in which there are no restrictions, but “the new normal” is popularly used to mean life with social distancing measures imposed. I suggest using a more precise description like “the baseline reproduction number in the absence of interventions”.

We avoid mentioning the “new normal” and just mention the basal r_0=1.8

The causality in this sentence is also the wrong way round: ri_no test = r0 because of the absence of travel restrictions, whereas the authors make it sound like the value of the reproductive ratio is what causes the absence of travel restrictions. The authors should make it clear that this parameter value reflects, rather than determines, reality.

We amended this sentence to: ri_no test = r0 no travel restrictions are necessary

Page 15, line 270-271: “The coefficient $\alpha(t) \in [0; 1]$ weighs the portion of the considered time t that corresponds to the time spent outside their home city, so $\alpha(t) = 1/3$”. I can not see anything suggesting that the value of this coefficient is 1/3, and I am not sure if the authors mean something more like “we will set $\alpha(t)=1/3$”. For publication, the authors should explain whether this value is estimated from some data or is chosen arbitrarily; given the challenges surrounding mobility data this assumption seems reasonable.

Out of the 24h we consider that approximately 2/3 are spent on work and commuting. Since we simulate the equations using an integration method with a step of one day, we consider alpha=⅓, thus, we are taking a weighted proportion between working and night hours.

On page 17, there is a section called “Table with parameters”, containing no text. I think this is meant to contain the parameter table but because of the way LaTex formats tables the table appears on the next page. The authors should delete this section heading, moving the table into another subsection and referencing it in the main body text.

We removed the section and now display the Table when discussing the model.
