# Supplementary material for: Smart testing and critical care bed sharing for COVID-19 control
Source: PLoS One. 2021 Oct 6;16(10):e0257235. doi: 10.1371/journal.pone.0257235 (PMC8494319; doi:10.1371/journal.pone.0257235)
Supplement: Attachment — Submitted filename: Response to Reviewers.pdf [file pone.0257235.s010.pdf]

12 July 2021

Dear Editor,

Thank you for your letter and the opportunity to revise our paper on ‘Smart testing and critical care bed sharing for COVID-19 control’. We are delighted that referees recommended the paper for publication and offered suggestions to improve its presentation. We have addressed this issue in the new version. We have included the reviewer’s comments immediately after this letter and responded to them individually.

Sincerely Yours

Tiago Pereira, PhD

Reviewer #1:

I thank the authors for making a genuine attempt to address all of the comments raised by each reviewer. In doing so, I agree that the paper has been considerably strengthened. However, I must remark that my original concern about the appropriateness of the model structure related to the effects of *both* asymptomatic *and* presymptomatic individuals. Whilst consideration of asymptomatics has been addressed through the updated text appearing in the main article, I still believe that the issue of presymptomatic infection remains unresolved. I am satisfied if this is left as a limitation of the study, but I still feel that its potential effects should be discussed – particularly given how prominent the lag between onset of infectiousness and onset of symptoms is for COVID-19, and how important this effect could be to any quarantine-based intervention.

We added a sentence in Line 260 discussing the role of presymptomatic individuals.

That aside, my remaining comments are relatively minor:

- In equation (3), is capital I the set of all regions?

  Yes. We included the explanation

- Line 200. Is it possible that the stabilizing case rate in Sao Paulo city was a result of test saturation (i.e., there were no more tests available each day to detect additional cases)?

  Sao Paulo city has better testing capabilities than the interior. As the available tests captured the peak in the interior, we presume that they are enough to uncover the peak in the city.

- Line 220. Is it possible to make this statement more precise? Are you saying that ICU bed sharing combined with smart testing is sufficient to ensure that ICU usage in the metropolitan area remains below capacity (as opposed to simply reducing it)?
- Figure 3. Forgive me for possibly misunderstanding, but would transferring even more ICU beds from Sao Paulo city across to the Metropolitan area reduce the number of excess beds required there in the "new normal" scenario (whilst still leaving sufficient beds in the city region)?

  These two points can be clarified together. When compared to the rest of the state, the ICU capacity in SP city is larger. For the considered stage of the spreading, the metropolitan area can afford sharing beds with other regions and ensure its ICU usage remains below capacity by performing smart testing. By contrast, without bed sharing, the other regions cannot prevent ICU saturation only by increasing the number of tests. This phenomenon is related to the different epidemiological times of SP city, where the spreading started earlier (SP international airports were the gate of entrance of coronavirus in Brazil). Regarding Figure 3, if SP city shares more beds, the situation in the rest of the state improves. However, one should keep in mind that beds are not physically transferred. Rather, patients are flought into SP city from other regions. The figure shows indeed some slack that could be used to increase the number of patients coming from the countryside that are taken by SP city. We presented a configuration that sheds light on the advantage of implementing a pool of beds in the whole state. But we do not claim the considered percentage is the best one. The exact percentage should be determined by public-health authorities keeping in mind that this is a dynamic process.

- Line 300: "decreasing" instead of "decreasint"; "multiplying" instead of "multiplibying"

  Done.

- The notation used in the Supplementary Note may need to be updated following the changes to the main article (e.g., the parameters r_t, \sigma and \zeta appear to have been superseded). Also is it possible to re-label \nu in the supplement as this symbol is also used to denote the length of stay in ICU in the main article.
  Done. We changed state variables to mathcal and r_t to R_t.

Reviewer #3:

I thank the authors for their response to the comments of myself and the other reviewers. Unfortunately I do not feel that the authors have sufficiently addressed all of my comments. In particular, the paper still lacks an explicit explanation of what their smart testing regime actually involves, and a clear definition and justification for the parameter alpha. I would also like to point out that the line references in the authors' response were incorrect, and I hope that in any further rounds of review they are careful to make sure this does not happen again.

Detailed notes: I am still not satisfied that the authors have given an explicit definition of the smart testing regimen. My understanding is that by smart testing, the authors mean solving an optimal control problem and conducting tests according to the solution to that optimal control problem. The newly added paragraph on page 22 talks a bit about the optimal control problem, but the paper as a whole still lacks any explicit statement that smart testing means solving that optimal control problem and conducting tests accordingly. The authors need to provide a definition of smart testing in the introduction, and ideally in the abstract. This definition does not need to involve any mathematical detail, but should let the reader know unambiguously that the smart testing regimen means distributing tests according to where the optimal control problem suggests they will have the largest impact.

We included the definition in the abstract.

At the bottom of page 7 and going over to page 8, the authors says that "when R^i_(no test)(t)=R_0, no travel restrictions are necessary". While, having read the paper a few times, I understand what the authors mean here, on first reading this statement is extremely counterintuitive. The basic reproductive ratio is the reproductive ratio of cases in a population undergoing "normal" behaviour with no population-level immunity. This means we almost always assume that the effective reproductive ratio will be smaller than the basic reproductive ratio, partly because NPIs seek to reduce social mixing, but also because even in the absence of NPIs the accumulation of population-level immunity will reduce the stock of susceptibles available to each case. This means that to get an effective reproductive ratio higher than R_0, we need either an ongoing intensification of social contacts, or rapid population growth exceeding the rate of accumulation of immunity. Of course, on reflection it is clear that since R^i(no test)(t) is a maximum value for effective reproductive ratio given some restraints, what the authors are saying is that if we can keep hospitalisations below 90% of capacity with an effective reproductive ratio equal to R_0, then we don't need to implement restrictions. I recommend that the authors add a statement to this effect, making it very explicit that their aim is to find the maximal reproductive ratio which keeps hospital occupancy below 90% of capacity. While I am aware I have already requested on notation change in this context, some confusion could also be avoided by using slightly different notation to avoid confusion between this maximum and the actual value of the effective reproductive ratio – for instance by adding a hat or tilde to R^i_(no_test)(t). The authers subsequently define R^i_test(t) to be the instantaneous observed reproductive ratio (not a maximum!) under a given testing regime, which amplifies this confusion and the need to explicitly distinguish between maxima and attained values.

We followed the recommendation added sentence in Line 130

Unfortunately, I am not convinced that the authors have sufficiently addressed my query regarding the parameter alpha, the proportion of time per day spent outside of an individual's resident location. The authors say "Out of the 24h we consider that approximately 2/3 are spent on work and commuting. Since we simulate the equations using an integration method with a step of one day, we consider alpha = 1/3,

thus, we are taking a weighted proportion between working and night hours." It is extremely unclear to me what this means. The first sentence appears to mean that 16 hours per day are spent outside of one's resident location; while the authors do not supply any reference to data, this seems like an overestimate. While I can imagine that in very economically deprived contexts individuals may indeed need to work away from home for 16 hours at a time, this seems very high for the time per day that an average individual spends away from their home location on an average day. This would also give alpha=2/3, based on the definition they give of alpha as the proportional amount of time spent outside of the household, rather than alpha=1/3. I am not sure what the authors mean by "a weighted proportion between working and night hours"; if they are treating "night" and "home" contacts as the same thing, I would recommend sticking to "home" as the terminology here. I note also that in Equation 6 on page 18 they appear to use alpha as the coefficient for within-patch mixing, in which case (by their assumption about commute/work time), alpha would indeed by 2/3. Again, in the description of Equation 6 they talk about "day and night" portions, and they either need to replace this with "work and home" or else state very clearly what their assumptions are about how work and home contacts are distributed over day and night. The authors need to check that their written definition of alpha actually corresponds to its role in the model, accompanied with a simple explanation of their choice of value along the lines of "we choose a value of alpha equal to 1/3 (or 2/3) on the assumption that on average individual spends 8 (or 16) hours per day away from their home location".

We apologize that our explanation was not sharp. Working from 8 am to 6 pm is normal in developing countries such as Brazil. This takes into account lunch break. Moreover, a 1.5h to 2h commute is common. This is equivalent to considering a total time of 16h away from home. Moreover, we are assuming that α is a periodic function modeling the bulk of individuals leaving home to work and returning home. Since commute is stereotyped and workers move in and out at well-defined times the dynamics is close to a square wave. We are assuming $\alpha(t) = 0$ when workers are away from home and 1 otherwise.

In view of Floquet Theory and we can consider an average of the function

$$< \alpha > \; = \frac{1}{T}\int_0^T \alpha(s)\, ds$$

where T stands for 24h. This leads to $< \alpha > \; = \frac{1}{3}$, since our integration step is one full day. The numerical implementation considers the daily average value of α.

Spelling/grammar/style notes:

Page 2: "In this situation, nonlinear effects resulting from the high degree of population mobility makes the decision of optimal test distribution a real challenge also because many individuals cannot isolate as they need to get income" – I realise the end of this sentence was added in response to another reviewer's comments, but I am afraid the wording here is rather confusing, and the note about economic factors may be better off in its own sentence.

The sentence was split.

Page 5: Incorrect date of "July 31, 2021" is still there!

Date is correct as it is a result of simulation starting from July 1 2020.

Caption to Figure 1 on page 7: "Thicker edges represent larger commuting." – this should say something like "Thicker edges represent more intensive commuting."

Fixed

Page 11: The notation τ appears to be used before it is defined.

We defined τwith words only in Line 215.

Figure 3 on page 13 still includes the phrase “new normal”, despite its removal from the rest of the paper.

We removed all “new normal” wording from the text.

Newly added paragraph on page 22 makes repeated reference to Equation 18; there is no Equation 18 in the paper and I think the authors mean to be referencing Equation 17 here.

Thanks. We corrected it.
