# Supplementary material for: Smart testing and critical care bed sharing for COVID-19 control
Source: PLoS One. 2021 Oct 6;16(10):e0257235. doi: 10.1371/journal.pone.0257235 (PMC8494319; doi:10.1371/journal.pone.0257235)
Supplement: Attachment — Submitted filename: Rebuttal_2021.08.24.pdf [file pone.0257235.s011.pdf]

Review Comments to the Author

Reviewer #3: I am pleased to say that the authors have addressed all the points from my previous set of comments, although the newly-added definition of the parameter tau needs to be revised for grammar and clarity. The complete sentence currently reads as follows: “The results reported in Figure 3 were obtained under the premise that susceptible individuals seek healthcare assistance and are tested by RT-PCR where $\tau = 1$ day after experiencing symptoms, where τ the number of days it takes for an infectious to seek assistance”. I think this sentence really needs to be split in two, along the lines of “We define τ to be the number of days it takes for an infectious individual to seek assistance. The results reported in Figure 3 were obtained under the premise that susceptible individuals seek healthcare assistance and are tested by RT-PCR on average one day after experiencing symptoms, so that $\tau = 1$”.

My only other comment (and I apologise for not catching this on the first draft) is that the authors switch between using SEIQR and SEIRQ to denote their compartmental structure in their methods section. This should be corrected so that one acronym is used consistently.

Pending these two corrections, the manuscript is entirely suitable for publication.

We addressed the issues raised by the reviewer.
