# Supplementary material for: Smart testing and critical care bed sharing for COVID-19 control
Source: PLoS One. 2021 Oct 6;16(10):e0257235. doi: 10.1371/journal.pone.0257235 (PMC8494319; doi:10.1371/journal.pone.0257235)
Supplement: Attachment — Submitted filename: plos_review.docx [file pone.0257235.s009.docx]

I thank the authors for making a genuine attempt to address all of the comments raised by each reviewer. In doing so, I agree that the paper has been considerably strengthened. However, I must remark that my original concern about the appropriateness of the model structure related to the effects of *both* asymptomatic *and* presymptomatic individuals. Whilst consideration of asymptomatics has been addressed through the updated text appearing in the main article, I still believe that the issue of presymptomatic infection remains unresolved. I am satisfied if this is left as a limitation of the study, but I still feel that its potential effects should be discussed – particularly given how prominent the lag between onset of infectiousness and onset of symptoms is for COVID-19, and how important this effect could be to any quarantine-based intervention.

That aside, my remaining comments are relatively minor:

- In equation (3), is capital I the set of all regions?
- Line 200. Is it possible that the stabilizing case rate in Sao Paulo city was a result of test saturation (i.e., there were no more tests available each day to detect additional cases)?
- Line 220. Is it possible to make this statement more precise? Are you saying that ICU bed sharing combined with smart testing is sufficient to ensure that ICU usage in the metropolitan area remains below capacity (as opposed to simply reducing it)?
- Figure 3. Forgive me for possibly misunderstanding, but would transferring even more ICU beds from Sao Paulo city across to the Metropolitan area reduce the number of excess beds required there in the "new normal" scenario (whilst still leaving sufficient beds in the city region)?
- Line 300: "decreasing" instead of "decreasint"; "multiplying" instead of "multiplibying"
- The notation used in the Supplementary Note may need to be updated following the changes to the main article (e.g., the parameters r_t, \sigma and \zeta appear to have been superseded). Also is it possible to re-label \nu in the supplement as this symbol is also used to denote the length of stay in ICU in the main article.
